# Investigating the relationship between social media exposure, body image dissatisfaction, and self-compassion in adolescent athlete

**DOI:** 10.3389/fpsyg.2026.1819872

**Published:** 2026-06-05

**Authors:** Jie Chen, Tao Tao

**Affiliations:** 1 Huzhou Vocational &Technical College, Huzhou, Zhejiang, China; 2 Institute of Physical Education, Huzhou Normal University, Huzhou, Zhejiang, China

**Keywords:** adolescent athletes, body image dissatisfaction, fear of negative evaluation, self-compassion, Self-Compassion Theory, Social Comparison Theory, social media exposure

## Abstract

**Introduction:**

Social media exposure has become a significant influence on adolescents’ body image perceptions, particularly among adolescent athletes who face both performance-related and appearance-related pressures. This study investigated the relationship between social media exposure and body image dissatisfaction, focusing on the mediating role of self-compassion and the moderating role of fear of negative evaluation.

**Methods:**

A cross-sectional survey was conducted among 449 adolescent athletes in China. Data were collected using standardized questionnaires measuring social media exposure, self-compassion, body image dissatisfaction, and fear of negative evaluation. Structural equation modeling (SmartPLS 3.3.3) was employed to test the hypothesized mediation and moderation effects.

**Results:**

Social media exposure was positively associated with body image dissatisfaction and negatively associated with self-compassion. Self-compassion significantly mediated the relationship between social media exposure and body image dissatisfaction. Furthermore, fear of negative evaluation significantly moderated the relationship between social media exposure and self-compassion, such that the negative association was stronger among individuals with higher levels of fear of negative evaluation.

**Discussion:**

The findings suggest that social media exposure contributes to body image dissatisfaction among adolescent athletes both directly and indirectly through reduced self-compassion. Fear of negative evaluation further increases vulnerability to these effects. The study extends Social Comparison Theory and Self-Compassion Theory by integrating protective and vulnerability factors within a single framework and highlights the importance of interventions aimed at strengthening self-compassion and reducing excessive evaluative pressure to promote adolescent wellbeing.

## 1 Introduction

The rise of social media platforms has profoundly altered the way adolescents perceive and evaluate themselves. With visually driven content dominating digital spaces, adolescents—particularly those engaged in competitive sports—are increasingly exposed to appearance-focused imagery and idealized portrayals of bodies (Hardie et al., 2022). Such exposure provides fertile ground for social comparisons that may undermine body satisfaction and heighten vulnerabilities associated with adolescence. Social Comparison Theory (Festinger, 1954) provides a useful lens to understand these processes, positing that individuals evaluate themselves by comparing with others, particularly when objective standards are absent. For adolescent athletes, who already face pressures to maintain performance-oriented physiques, exposure to highly curated images on social media is likely to intensify upward comparisons and foster body image dissatisfaction (Bjornsen and O’Connor, 2025; Hilkens et al., 2021).

Body image dissatisfaction has long been identified as a key risk factor for low self-esteem, unhealthy dieting, and maladaptive psychological outcomes among adolescents (Li et al., 2024). Within sporting contexts, where body functionality and appearance are central, dissatisfaction may impair performance and overall wellbeing. Research has demonstrated that adolescent athletes are not immune to such concerns; in fact, pressures within both sport and media environments can exacerbate vulnerability (Zaccagni and Gualdi-Russo, 2023).

Although adolescent athletes are often perceived as physically fit, confident, and socially admired, accumulating empirical evidence challenges the assumption that athletic participation uniformly protects against body image concerns. Comparative studies consistently indicate that adolescent athletes report levels of body dissatisfaction that are comparable to, and in some cases exceed, those of non-athlete peers. For example, Di Corrado et al. (2021) demonstrated that athletes participating in appearance- and weight-sensitive sports exhibited heightened dissatisfaction relative to non-athletes, while Faria et al. (2021) reported no systematic protective effect of sport participation once sport-specific appearance pressures were considered. More recently, Webb et al. (2024) showed that adolescents engaged in sports emphasizing leanness or muscularity experienced elevated body surveillance and dissatisfaction comparable to non-athletes exposed to strong sociocultural appearance ideals. Taken together, prior literature suggests that sport participation may coexist with, rather than offset, appearance-related pressures, as athletic contexts introduce performance-linked body monitoring, coach and peer evaluation, and sport-specific aesthetic norms (Bjornsen and O’Connor, 2025; Cusack et al., 2022).

Moreover, sport participation may introduce unique appearance-related pressures, including performance-linked body surveillance, coach and peer evaluation, and sport-specific aesthetic norms, which coexist with broader sociocultural beauty ideals reinforced through social media (Hardie et al., 2022; Zaccagni and Gualdi-Russo, 2023). Social media exposure, therefore, may function not merely as a passive activity but as a contextual influence associated with identity construction and body evaluations in adolescence (Young et al., 2022). These processes are particularly salient in collectivist societies such as China, where peer perceptions and group evaluations strongly influence adolescents’ self-concepts (Li et al., 2024; Lin et al., 2025). Importantly, within the Chinese context, such peer- and group-based evaluations are closely tied to future-oriented concerns, including academic achievement, athletic performance legitimacy, and long-term educational or career prospects. For adolescent athletes, perceptions of physical competence and appearance often become symbolic markers of discipline, success, and future opportunity, thereby linking body-related evaluations to broader achievement and life-course expectations rather than to physical satisfaction alone (Lin et al., 2025; Zaccagni and Gualdi-Russo, 2023).

Despite the evidence linking media exposure and body dissatisfaction, protective psychological resources remain less examined in athlete populations. Self-Compassion Theory (Neff, 2003) provides an explanatory framework to address this gap. Self-compassion involves treating oneself with kindness during failure or imperfection, recognizing one’s experiences as part of common humanity, and holding a mindful, nonjudgmental stance toward personal shortcomings. In the context of social media exposure, self-compassion is theorized to counteract the damaging effects of upward comparisons by fostering acceptance and reducing harsh self-criticism (Balbinotti, 2025). Empirical research has shown that higher self-compassion is associated with greater body appreciation and lower levels of body dissatisfaction (Manjanatha et al., 2025). For adolescent athletes, cultivating self-compassion may buffer against negative body evaluations by shifting the focus from external appearance to intrinsic self-worth and psychological resilience (Neff, 2003; Mahon and Hevey, 2023; Hooper et al., 2024).

At the same time, the study also predicts that individual vulnerabilities can exacerbate the influence of social media on self-perception. Fear of negative evaluation (Gabrys and Wontorczyk, 2023) reflects apprehension about being judged unfavorably by others and is particularly relevant in adolescence, a developmental stage characterized by heightened sensitivity to peer evaluation. Athletes with high fear of negative evaluation may be more susceptible to internalizing negative comparisons because they place greater weight on external judgments and approval. Recent studies suggest that fear of negative evaluation may strengthen the relationship between social comparison and negative psychological outcomes, making it a critical moderator in the pathway from social media exposure to body dissatisfaction (Salazar-Ayala et al., 2021).

Taken together, this study seeks to extend existing literature by testing a mediation model with first-stage moderation grounded in Social Comparison Theory and supported by Self-Compassion Theory. Specifically, the study investigates whether social media exposure predicts body image dissatisfaction among adolescent athletes through reduced self-compassion, and whether this mediated relationship is conditioned by fear of negative evaluation. By focusing on a Chinese adolescent athlete sample, the research also addresses contextual considerations, as collectivist cultural values may heighten sensitivity to peer approval and thus intensify the proposed relationships (Lin et al., 2025).

More importantly, the study argues that adolescent athletes experience a distinct form of dual evaluative pressure that differs from ordinary adolescent populations, as they are simultaneously exposed to sport-performance expectations and media-driven appearance ideals (Bjornsen and O’Connor, 2025; Webb et al., 2024). Unlike non-athlete adolescents, athletes are frequently evaluated not only for physical attractiveness but also for body functionality, discipline, and competitive legitimacy, thereby creating overlapping online and offline comparison pressures that may intensify body image vulnerability (Hardie et al., 2022; Zaccagni and Gualdi-Russo, 2023). In collectivist contexts such as China, these pressures may become even more salient because peer approval, coach authority, parental expectations, and collective reputation are closely tied to adolescents’ self-evaluations and future-oriented achievement concerns (Lin et al., 2025). From this perspective, fear of negative evaluation represents more than a general social anxiety construct; rather, it reflects a culturally reinforced sensitivity to external judgment that may amplify the harmful psychological consequences of appearance-focused social media exposure among adolescent athletes (Gabrys and Wontorczyk, 2023). Accordingly, the present study contributes to the literature by positioning body image dissatisfaction in adolescent athletes as a contextually embedded process shaped through the interaction of social comparison pressures, compassionate self-regulation, and culturally heightened evaluative sensitivity.

The findings are expected to contribute both theoretically and practically, offering insights int. mechanisms that underlie body image concerns and identifying psychological resources that can mitigate risks in adolescent sports populations.

## 2 Literature review

Social media exposure has been increasingly recognized as a powerful influence on adolescents’ psychological development. As digital environments saturate daily life, adolescents spend considerable time on platforms that prioritize image-sharing and appearance-based interactions. According to Social Comparison Theory (Festinger, 1954), individuals assess themselves by comparing with others, and adolescents are particularly vulnerable because they are still consolidating identity and self-worth. A growing body of research confirms that increased exposure to social networking sites fosters upward comparisons with idealized images, which, in turn, predict negative outcomes such as body dissatisfaction and lower self-esteem (Di Corrado et al., 2021). Within sports contexts, adolescent athletes face not only external comparisons on social media but also performance-driven appearance norms in their disciplines, making them especially susceptible to dissatisfaction when confronted with unrealistic ideals online (Şentürk and Gobel, 2023).

On the other hand, body image dissatisfaction has been consistently linked to maladaptive outcomes in adolescence. Classic reviews emphasize that dissatisfaction predicts unhealthy eating, depressive symptoms, and diminished self-confidence (Li et al., 2024). More recent studies confirm that dissatisfaction is not confined to non-athletes; competitive athletes, particularly those in aesthetic sports such as gymnastics or dance, report high levels of dissatisfaction despite their active lifestyles (Bethel, 2024; Faria et al., 2021). The role of social media in this process has become especially salient, as the continuous stream of curated bodies online provides constant benchmarks for evaluation. Research highlights that such dissatisfaction not only undermines wellbeing but may also disrupt athletic performance by shifting focus from functional ability to appearance concerns (Hardie et al., 2022; Zaccagni and Gualdi-Russo, 2023). These findings highlight the importance of examining mechanisms that either exacerbate or buffer these effects.

One such mechanism is self-compassion, a construct that has gained considerable attention as a protective psychological resource. Self-Compassion Theory (Neff, 2003) conceptualizes the construct as encompassing three interrelated components: self-kindness versus self-criticism, common humanity versus isolation, and mindfulness versus over-identification. Empirical studies demonstrate that self-compassion is associated with lower levels of body dissatisfaction, higher body appreciation, and reduced internalization of sociocultural appearance ideals (Cusack et al., 2022). For adolescents, self-compassion appears to serve as a buffer against the harmful effects of appearance-based social comparisons (Kit et al., 2025). Among athletes, self-compassion has also been shown to protect against self-criticism in response to performance setbacks and to foster resilience in the face of evaluative pressures (Tylka and Huellemann, 2023). This evidence suggests that lower self-compassion may explain why adolescents exposed to high levels of social media experience greater body dissatisfaction.

**Figure 1:**
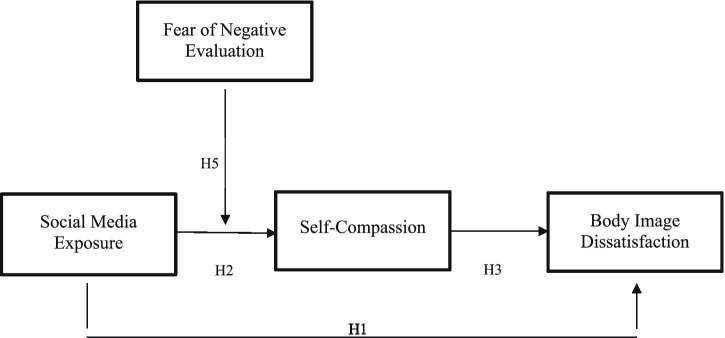
Conceptual model.

At the same time, vulnerabilities such as fear of negative evaluation may amplify these processes. Fear of negative evaluation, originally defined by Watson and Friend (1969), captures apprehension about being judged unfavorably by others and has since been established as a central feature of social anxiety. Adolescents with high fear of negative evaluation are particularly concerned with external judgments, which makes them more sensitive to the evaluative nature of social media environments. Past studies have demonstrated that fear of negative evaluation intensifies the impact of social comparison on negative affect, body concerns, and self-esteem (Salazar-Ayala et al., 2021; Weeks et al., 2005). For athletes, whose performance and appearance are regularly scrutinized by coaches, peers, and audiences, fear of negative evaluation may create a heightened vulnerability to both sport-related and media-related pressures. In this sense, fear of negative evaluation functions as a moderator, conditioning the extent to which social media exposure erodes self-compassion and fuels dissatisfaction. Bringing these strands together, prior literature suggests that body image dissatisfaction among adolescent athletes emerges at the intersection of social comparison processes, protective resources such as self-compassion, and vulnerabilities such as fear of negative evaluation (Figure 1).

### 2.1 Hypotheses development

#### 2.1.1 Linking social media exposure with body image dissatisfaction

The study posits that social media exposure exerts a direct influence on body image dissatisfaction among adolescent athletes. Drawing from Social Comparison Theory, Festinger (1954) argued that individuals naturally evaluate themselves in relation to others when objective benchmarks are absent, and visual-based platforms can amplify this tendency under conditions where appearance-focused and evaluative content is salient, as such environments increase the accessibility of idealized portrayals of beauty and physique and facilitate upward comparison processes (Vandenbosch et al., 2022; Jiotsa et al., 2021). Research consistently demonstrates that increased engagement with social networking sites predicts higher levels of body dissatisfaction, particularly when individuals are exposed to appearance-related content (Klier et al., 2022). In addition, a systematic review concluded that social networking site use reliably correlates with negative body evaluations across adolescent and young adult samples, underscoring the robustness of this association (Li et al., 2024). Another stream of research highlights the unique vulnerability of athletes, who often face dual pressures from both sports-related appearance ideals and broader societal beauty standards. For example, Beos et al. (2025) found that adolescent athletes in aesthetic sports reported elevated dissatisfaction, a finding echoed by Webb et al. (2024), who noted that online exposure exacerbates these concerns. More recently, meta-analytic evidence has confirmed that upward appearance comparisons made in digital contexts intensify negative affect and dissatisfaction (Zaccagni and Gualdi-Russo, 2023). Collectively, these findings support the expectation that adolescents exposed to high volumes of social media content are more likely to experience dissatisfaction with their bodies.

*H1*: The study posits that social media exposure is positively associated with body image dissatisfaction among adolescent athletes.

#### 2.1.2 Linking social media exposure with self-compassion

The study further anticipates that social media exposure undermines adolescents’ ability to practice self-compassion. Whereas Social Comparison Theory emphasizes the tendency to contrast oneself against superior standards (Festinger, 1954), Self-Compassion Theory (Neff, 2003) highlights the role of kindness, mindfulness, and common humanity in counteracting such harsh self-evaluations. When exposure to idealized imagery becomes frequent, adolescents are more likely to internalize upward comparisons, which can erode compassionate self-attitudes. In a longitudinal investigation, Scott et al. (2021) observed that higher social networking site use predicted lower self-acceptance over time, suggesting that the process of comparison gradually weakens self-directed care. Other scholars point out that adolescents who engage in appearance-based browsing report greater self-criticism, a tendency directly opposed to the self-kindness component of self-compassion (Staśkiewicz-Bartecka et al., 2024). Another thread of research in clinical and non-clinical settings further shows that self-compassion is inversely related to the degree of internalization of media ideals, suggesting that digital environments shape self-perception through reduced mindful acceptance (Bjornsen and O’Connor, 2025). Moreover, cross-cultural evidence indicates that adolescents in collectivist societies are particularly sensitive to external evaluations, which magnifies the negative effect of media exposure on compassionate self-attitudes (Izydorczyk et al., 2023). Based on these findings, it is reasonable to expect that increased exposure to social media platforms will be associated with diminished self-compassion among adolescent athletes.

*H2*: The study anticipates that social media exposure is negatively associated with self-compassion among adolescent athletes.

#### 2.1.3 Linking self-compassion with body image dissatisfaction

Subsequently, adolescents who cultivate self-compassion often demonstrate more balanced evaluations of their bodies and are less likely to internalize rigid standards of beauty. Prior research indicates that individuals high in self-compassion display greater body appreciation and lower levels of disordered eating behaviors (Li et al., 2025; Seekis, 2020). In addition, an experimental study by Hooper et al. (2024) showed that interventions designed to enhance self-compassion significantly reduced body dissatisfaction and shame. Another line of evidence highlights that self-compassion buffers the emotional costs of negative appearance comparisons, thereby mitigating the detrimental effects of media exposure (Mahon and Hevey, 2023). Meta-analytic findings further support these conclusions, reporting consistent negative associations between self-compassion and body image concerns across adolescent and young adult samples (Linardon et al., 2022). These outcomes converge to suggest that adolescents who practice self-kindness, recognize their struggles as part of shared human experience, and maintain mindful awareness of self-criticism are less likely to develop body dissatisfaction.

Building on this perspective, evidence increasingly suggests that self-compassion may serve as a bridge linking social media exposure and body image dissatisfaction. For example, Tylka and Huellemann (2023) observed that self-compassion mediated the effect of internalized appearance ideals on disordered eating, pointing to its central role in translating external pressures into internal distress. In another investigation, Adam et al. (2021) demonstrated that individuals high in self-compassion were less negatively affected by thin-ideal media, indicating that compassion dampens the pathway from media exposure to dissatisfaction. A systematic review has also emphasized that adolescents with stronger self-compassion experience fewer negative psychological consequences of online comparison, thereby reducing the risk of dissatisfaction (Beos et al., 2025).

While these studies establish the protective function of self-compassion, they have largely focused on general adolescent or adult samples and have rarely examined adolescent athletes embedded in highly evaluative sport environments (Adam et al., 2021; Tylka and Huellemann, 2023). Moreover, prior research has typically tested direct buffering or simple mediation effects, leaving unanswered questions about the contextual conditions under which self-compassion operates as a mechanism linking social media exposure to body image outcomes (Beos et al., 2025; Linardon et al., 2022). By examining adolescent athletes in China and integrating fear of negative evaluation as a boundary condition, the present study extends this literature by specifying when and how self-compassion transmits the effects of social media exposure on body image dissatisfaction (Gabrys and Wontorczyk, 2023; Lin et al., 2025). These findings align with the expectation that adolescent athletes who spend more time on social media are likely to experience reduced self-compassion (Neff, 2003), which in turn increases their vulnerability to body dissatisfaction. In theoretical terms, Social Comparison Theory (Festinger, 1954) provides the basis for understanding how exposure to idealized online content heightens comparison processes, while Self-Compassion Theory clarifies how compassionate attitudes mediate the self-critical responses that lead to dissatisfaction. Integrating both perspectives, the mediating role of self-compassion represents a mechanism through which social media exposure translates into heightened body image concerns. Accordingly, the study anticipates that:

*H3*: The study proposes that self-compassion is negatively associated with body image dissatisfaction among adolescent athletes.

*H4*: The study anticipates that self-compassion mediates the relationship between social media exposure and body image dissatisfaction among adolescent athletes.

#### 2.1.4 Moderating effect of fear of negative evaluation

Moreover, adolescents who are highly concerned with how they are judged by others are particularly sensitive to evaluative environments, and social media represents a space that is inherently evaluative in nature. Fear of negative evaluation has been widely recognized as a central feature of social anxiety, with earlier work by Watson and Friend (1969) demonstrating its influence on how individuals perceive and internalize social judgments. More recent research confirms that adolescents with higher fear of negative evaluation are more reactive to appearance-related comparisons, reporting heightened distress and reduced confidence in their self-image (Li et al., 2025). In another stream of study, Lin et al. (2025) found that fear of negative evaluation intensifies the psychological consequences of social comparisons, suggesting that those with higher levels of this trait are especially vulnerable to dissatisfaction when exposed to evaluative cues. These findings are reinforced by Gabrys and Wontorczyk (2023), who argued that fear of negative evaluation serves as a unifying construct within social anxiety research, consistently predicting heightened sensitivity to perceived judgment.

Within the current study, such evidence implies that fear of negative evaluation not only exacerbates the direct link between social media exposure and body dissatisfaction but also shapes the pathway through self-compassion. Adolescents with high levels of this fear are more likely to respond to media-driven comparisons with harsh self-criticism (Fasbender and Gerpott, 2022), thereby undermining their capacity for self-kindness and acceptance. As a result, the negative association between social media exposure and self-compassion is expected to be stronger for these individuals, which, in turn, amplifies their risk of developing dissatisfaction. Conceptually, fear of negative evaluation operates at the social appraisal stage by heightening sensitivity to external judgments and comparisons (Watson and Friend, 1969; Weeks et al., 2005), whereas self-compassion represents an intrapersonal regulatory resource that governs how such inputs are processed and transformed into self-evaluations (Neff, 2003; Muris et al., 2016). Accordingly, once self-compassion is activated, downstream effects on body image dissatisfaction are less contingent on evaluative fear, providing a theoretical rationale for specifying moderation at the initial exposure-to-self-compassion link rather than at subsequent paths. From a theoretical perspective, Social Comparison Theory (Festinger, 1954) explains how evaluative contexts fuel comparison processes, while Self-Compassion Theory (Neff, 2003) clarifies why low levels of compassionate self-regulation make adolescents more vulnerable. Integrating these perspectives, the study proposes that:

*H5*: Fear of negative evaluation moderates the relationship between social media exposure and self-compassion, such that the negative effect is stronger when fear of negative evaluation is high.

## 3 Research methodology

This study employed a cross-sectional explanatory design using variance-based structural equation modeling (PLS-SEM) to test the direct, mediated, and moderated relationships among social media exposure, self-compassion, body image dissatisfaction, and fear of negative evaluation in adolescent athletes. Data were collected once through standardized self-report scales printed in Chinese and administered in natural school and team settings. A multistage sampling approach was used, involving cluster selection of sports-focused middle and high schools and community training centers across three provinces in China, followed by proportionate stratification by sport type (aesthetic vs. non-aesthetic) and school level (middle vs. high). Within each region, one to two provinces were purposively selected based on the presence of established youth sports programs, and official lists of sports-specialized schools and registered training centers served as the sampling frame. Within each stratum, intact classes and teams were approached, and all eligible athletes present during data collection were invited to participate.

A total of 500 paper questionnaires were distributed during scheduled training sessions, of which 472 were returned, yielding a crude response rate of 94.4 percent. After screening for missing values, patterned responding, and ineligibility, 449 cases were retained for final analysis. Participants were adolescent athletes actively engaged in organized training programs and competing at least twice weekly. Specifically, 500 athletes were approached, 472 met the eligibility criteria and consented to participate, and 23 responses were excluded due to substantial missing data or response patterns indicative of low engagement, resulting in a final analytic sample of 449. Detailed sample characteristics—including gender, age group, school level, sport type, training experience, weekly training hours, daily social media use, and regional distribution—are summarized in Appendix 1.

Fieldwork was conducted between March and May 2025 over a period of 10 weeks. Trained research assistants collaborated with coaches and school administrators to administer printed questionnaires during team practices or classroom periods. Surveys were completed on site under supervision and returned immediately in sealed envelopes, with make-up sessions provided within a week for absentees. To mitigate potential common method bias during data collection, several procedural remedies were implemented, including ensuring respondent anonymity, emphasizing that there were no right or wrong answers, and minimizing evaluation apprehension by clarifying that responses would be used solely for research purposes. The final analytic sample size of 449 exceeded recommended minimum thresholds for PLS-SEM, ensuring adequate statistical power for estimating direct, mediating, and moderating effects.

Prior to structural model estimation, data normality was assessed through skewness and kurtosis statistics. Several indicators exceeded the commonly recommended thresholds for multivariate normality, suggesting mild to moderate non-normality within the dataset. Accordingly, PLS-SEM was considered appropriate because it is less sensitive to distributional assumptions and is particularly suitable for estimating complex moderated mediation models involving latent constructs and interaction effects (Hair et al., 2021). In addition, the study aimed not only to test theoretically grounded relationships but also to maximize explained variance and predictive relevance in body image dissatisfaction among adolescent athletes, further supporting the use of PLS-SEM in the present context.

Subsequently, descriptive statistics and bivariate correlations were examined to explore associations between key study variables and demographic characteristics such as sport type, training duration, and weekly training hours. In addition, preliminary group comparisons were conducted using independent-samples t-tests and one-way analyses of variance (ANOVAs) to examine whether key demographic variables (e.g., sport type and school level) were systematically related to the primary study constructs. These variables were not included as control variables in the structural model, as preliminary analyses indicated no systematic confounding effects on the hypothesized relationships. Specifically, preliminary correlation analyses, independent-samples t-tests, and one-way ANOVAs indicated that demographic variables such as gender, school level, and sport type did not produce consistent or substantively meaningful changes in the hypothesized relationships or path estimates. For transparency, a summary of these preliminary analyses has been reported in Appendix 4. Consistent with recommendations in PLS-SEM, control variables that do not meaningfully influence substantive relationships were excluded to avoid unnecessary model complexity and over-adjustment.

### 3.1 Measures

The study adapted scale items from previous studies mentioned in the subsequent sub-section. Across all measures, composite scores were computed using item means. Higher composite scores indicated higher levels of the respective constructs, and no items were reverse-coded. All instruments were translated into Chinese using a standard forward–back translation procedure, with discrepancies resolved through expert review. All items were measured using positively worded statements, and no reverse-coded items were included in the final analysis. Full item wording and scale details are provided in Appendix 2.

#### 3.1.1 Social media exposure

Social media exposure was measured using seven items adapted from established scales capturing the frequency and intensity of adolescents’ engagement with social networking sites (Appel et al., 2016; Meshi et al., 2015). Responses were recorded on a five-point Likert scale ranging from 1 (strongly disagree) to 5 (strongly agree). A sample item is: *“I often compare myself with others on social media.”* In the present study, social media exposure is conceptualized as adolescents’ engagement with appearance-oriented social media environments, including the frequency and intensity of interaction with image-based and evaluative content. Although the construct primarily reflects exposure and engagement, one item also captures appearance-related comparison behavior on social media, indicating that the measure partially reflects cognitively evaluative engagement with social media environments in addition to exposure intensity alone.

#### 3.1.2 Self-compassion

Self-compassion was assessed with eight items reflecting kindness toward oneself and balanced emotional awareness, adapted from Neff (2003). The abbreviated and validated adaptations of this measure have been widely applied in adolescent populations (Neff and Germer, 2017; Muris et al., 2016). Responses were rated on a five-point Likert scale from 1 (almost never) to 5 (almost always). A sample item is: *“I try to be understanding and patient toward aspects of my personality I do not like.”*

#### 3.1.3 Body image dissatisfaction

Body image dissatisfaction was measured with nine items reflecting adolescents’ negative evaluations of their body shape and appearance (Cash, 2000; Mutale et al., 2016). Items were rated on a five-point Likert scale ranging from 1 (strongly disagree) to 5 (strongly agree). A sample item is: *“I often feel unhappy with the way my body looks.”*

#### 3.1.4 Fear of negative evaluation

Fear of negative evaluation was assessed with five items adapted from the Brief Fear of Negative Evaluation Scale (Leary, 1983). This measure has been widely validated across adolescent and athlete populations in recent studies (Meyers, 2023; Weeks et al., 2005). Participants responded on a five-point Likert scale from 1 (not at all characteristic of me) to 5 (extremely characteristic of me). A sample item is: *“I worry about what other people think of me even when I know it does not matter.”*

## 4 Results

Partial Least Squares Structural Equation Modeling (PLS-SEM) version 3.3.3 was employed to evaluate both the measurement and structural models. PLS-SEM is considered suitable when models are complex, involve latent constructs with multiple indicators, and focus on prediction and theory development rather than strict model fit (Hair et al., 2021). It is particularly effective for smaller to medium sample sizes and non-normal data distributions, making it widely adopted in social sciences and psychology research (Chin, 1998; Hair et al., 2021). Using SmartPLS 3.3.3 thus provided a robust platform to test direct, indirect, and moderating effects in the present model.

The measurement model was first assessed using outer loadings (Table 1). Outer loadings above 0.70 are generally considered satisfactory, although items between 0.40 and 0.70 can be retained if they contribute to content validity and the composite reliability of the construct (Hair et al., 2021). In this study, body image dissatisfaction items loaded between 0.738 and 0.823, demonstrating strong reliability. Fear of negative evaluation items ranged from 0.609 to 0.874, with one item slightly below 0.70 yet acceptable in exploratory settings (Hulland, 1999). Self-compassion items demonstrated loadings between 0.761 and 0.841, while social media exposure items ranged from 0.613 to 0.806, again confirming satisfactory measurement quality. These findings support indicator reliability across constructs.

**Table 1:** Measuring indicators’ reliability using outer loadings.

Indicators	Body image dissatisfaction	Fear of negative evaluation	Self-compassion	Social media exposure
BID1	0.746			
BID2	0.738			
BID3	0.775			
BID4	0.796			
BID5	0.746			
BID6	0.792			
BID7	0.79			
BID8	0.772			
BID9	0.823			
FNE1		0.874		
FNE2		0.842		
FNE3		0.797		
FNE4		0.676		
FNE5		0.609		
SC1			0.799	
SC2			0.772	
SC3			0.789	
SC4			0.804	
SC5			0.806	
SC6			0.841	
SC7			0.8	
SC8			0.761	
SME2				0.802
SME3				0.79
SME4				0.783
SME5				0.806
SME6				0.755
SME7				0.613
SME1				0.793

Construct reliability and validity were then assessed (Table 2). Cronbach’s alpha values exceeded the recommended 0.70 threshold for all constructs, including social media exposure (*α* = 0.881), self-compassion (*α* = 0.918), fear of negative evaluation (*α* = 0.831), and body image dissatisfaction (*α* = 0.917), thereby confirming satisfactory internal consistency (Nunnally and Bernstein, 1994). Composite reliability scores were all above 0.70 and below the upper limit of 0.95, ensuring reliability without redundancy (Hair et al., 2021). The average variance extracted (AVE) values for all constructs exceeded the 0.50 benchmark (Fornell and Larcker, 1981), indicating convergent validity by capturing more than half of the variance of indicators in their latent constructs. These results collectively demonstrate strong construct reliability and validity. In addition to these variance-based PLS-SEM reliability and validity assessments, a supplementary confirmatory factor analysis was conducted using conventional covariance-based criteria. As reported in Appendix 3, the CFA demonstrated acceptable model fit (*χ*^2^/df = 2.18, CFI = 0.942, TLI = 0.931, RMSEA = 0.057, SRMR = 0.046), providing complementary evidence for the adequacy of the measurement model.

**Table 2:** Constructs’ reliability and validity.

Constructs	Cronbach’s alpha	rho_A	Composite reliability	Average variance extracted (AVE)
Body Image Dissatisfaction	0.917	0.919	0.931	0.602
Fear of Negative Evaluation	0.831	0.885	0.875	0.588
Self-Compassion	0.918	0.92	0.933	0.635
Social Media Exposure	0.881	0.887	0.908	0.587

Discriminant validity was tested using the HTMT criterion (Table 3). According to Henseler et al. (2015), HTMT values should remain below 0.85 for conservative models or 0.90 for more liberal thresholds. In this study, the highest HTMT value was 0.592 between self-compassion and body image dissatisfaction, which remained well below the 0.85 threshold. Similarly, other HTMT values such as 0.528 between social media exposure and body image dissatisfaction and 0.509 between social media exposure and self-compassion also fell below the cut-off. These results confirm that the constructs are empirically distinct and that discriminant validity is established.

**Table 3:** Discriminant validity.

Constructs	Body image dissatisfaction	Fear of negative evaluation	Self-compassion	Social media exposure
Body image dissatisfaction				
Fear of negative evaluation	0.062			
Self-compassion	0.592	0.111		
Social media exposure	0.528	0.086	0.509	

Collinearity was examined through variance inflation factors (VIF) (Table 4). VIF values below 5 indicate the absence of multicollinearity concerns (Hair et al., 2021). All predictors in this study had VIF values between 1.024 and 1.269, comfortably within the recommended threshold. This suggests that the predictors contributed unique variance to the model without problematic overlap, allowing reliable estimation of path coefficients. In addition, potential common method bias was assessed using Harman’s single-factor test. The unrotated factor solution revealed that the first factor accounted for 39.6 percent of the total variance, indicating that common method bias was unlikely to pose a serious threat to the validity of the findings. Although Harman’s single-factor test has recognized limitations, the results, combined with the procedural remedies implemented during data collection, suggest that common method bias is unlikely to have substantially influenced the observed relationships.

**Table 4:** Collinearity statistics (VIF).

Predictor	Endogenous construct	VIF
Social media exposure	Self-compassion	1.024
Fear of negative evaluation	Self-compassion	1.024
Social media exposure	Body image dissatisfaction	1.269
Self-compassion	Body image dissatisfaction	1.269

The structural model results are summarized in Table 5. To assess the significance of path coefficients and indirect effects, a nonparametric bootstrapping procedure was applied in SmartPLS. Bootstrapping was conducted using 5,000 resamples, with bias-corrected and accelerated (BCa) confidence intervals, and the no sign changes option was applied. Hypotheses were evaluated based on whether the confidence intervals excluded zero.

**Table 5:** Structural path results.

Hypotheses	*B*	STDEV	*T*-value	*p* values	2.50%	97.50%
Social media exposure - > body image dissatisfaction	0.286	0.058	4.921	0.000	0.172	0.401
Social media exposure - > self-compassion	−0.436	0.068	6.446	0.000	−0.562	−0.303
Self-compassion - > body image dissatisfaction	−0.417	0.06	6.93	0.000	−0.545	−0.298
Social media exposure - > self-compassion - > body image dissatisfaction	0.182	0.041	4.387	0.000	0.108	0.272
Fear of negative evaluation - > self-compassion	0.046	0.020	2.312	0.036	0.027	0.123
Fear of negative evaluation x social media exposure - > self-compassion	−0.125	0.061	2.07	0.041	−0.225	−0.025

Social media exposure significantly and positively predicted body image dissatisfaction (*β* = 0.286, *p* < 0.001), aligning with research linking exposure to negative body perceptions among adolescents (Hardie et al., 2022; Zaccagni and Gualdi-Russo, 2023). Social media exposure also had a significant negative effect on self-compassion (*β* = −0.436, p < 0.001), supporting prior findings that media exposure undermines positive self-attitudes (Muris et al., 2016; Neff and Germer, 2017). In turn, self-compassion significantly reduced body image dissatisfaction (*β* = −0.417, *p* < 0.001), confirming its protective role as established in earlier literature (Cusack et al., 2022; Kit et al., 2025). The indirect effect of social media exposure on body image dissatisfaction through self-compassion was also significant (*β* = 0.182, *p* < 0.001), providing evidence of partial mediation. Finally, the interaction between fear of negative evaluation and social media exposure significantly predicted self-compassion (*β* = −0.125, *p* = 0.039). This finding indicates that fear of negative evaluation strengthened the negative association between social media exposure and self-compassion. Specifically, adolescents with higher levels of fear of negative evaluation experienced a steeper decline in self-compassion as social media exposure increased. Such moderation highlights the conditional nature of the indirect effect, suggesting that individuals with heightened sensitivity to external judgment may be particularly vulnerable to the adverse psychological consequences associated with appearance-oriented social media exposure. These findings not only provide statistical support for the proposed model but also extend theoretical understanding of how psychological vulnerabilities amplify the adverse effects of social media.

**Figure 2:**
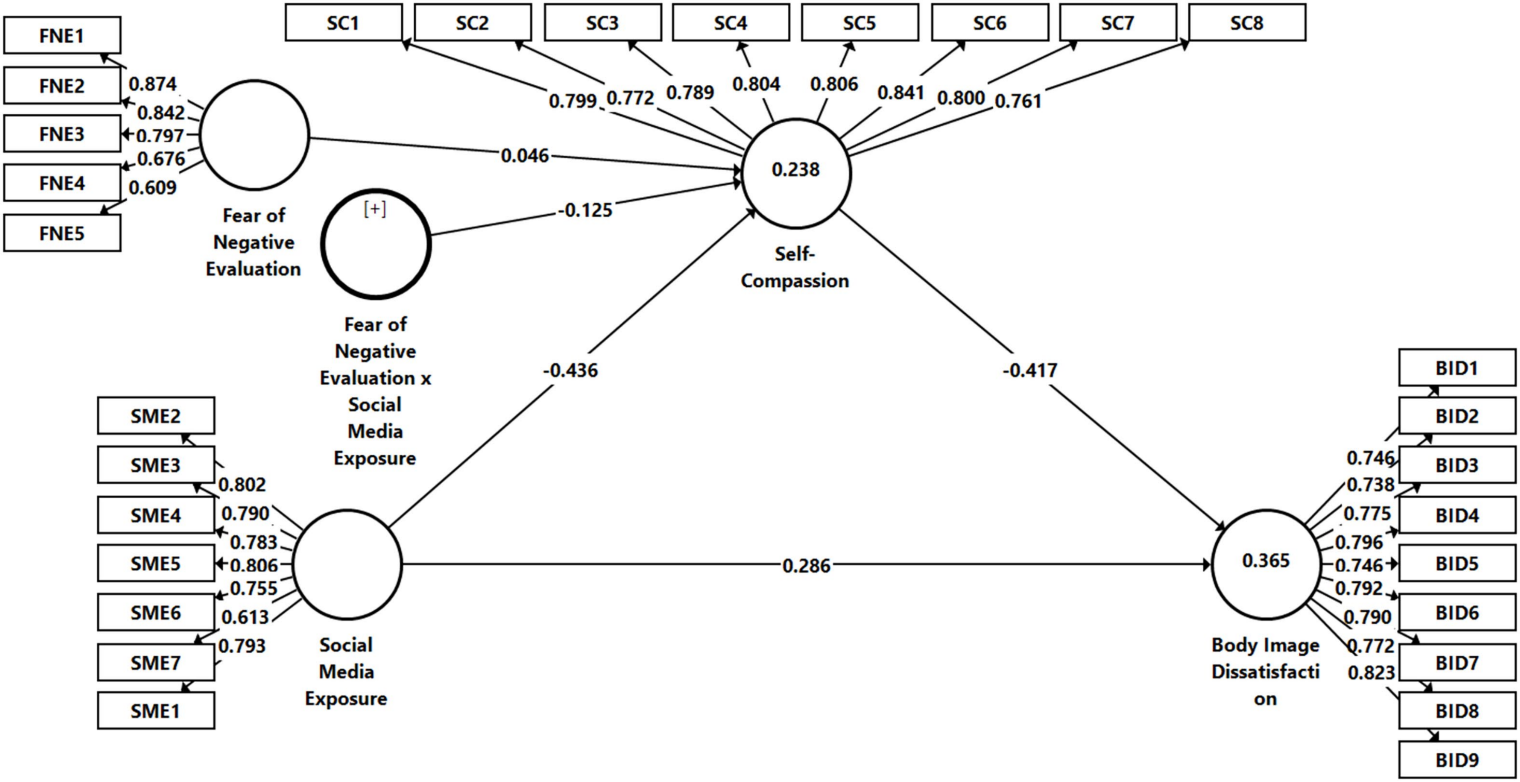
Structural model showing the relationships among social media exposure, self-compassion, fear of negative evaluation, and body image dissatisfaction among Chinese adolescent athletes. Standardized path coefficients are reported.

The results of this analysis are also illustrated in Figure 2. The figure illustrates the hypothesized relationships between social media exposure, self-compassion, body image dissatisfaction, and fear of negative evaluation. Direct, indirect, and moderated paths are displayed along with explained variance (*R*^2^) for endogenous constructs. In this model, the *R*^2^ values indicate that 23.8% of the variance in self-compassion and 36.5% of the variance in body image dissatisfaction were explained by the predictors, which reflects moderate explanatory power according to Hair et al. (2021).

**Figure 3:**
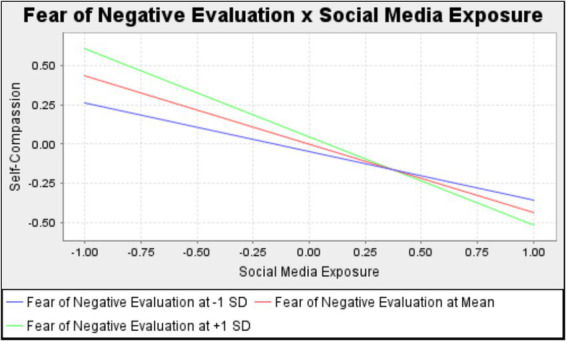
Moderating effect of fear of negative evaluation on the relationship between social media exposure and self-compassion among Chinese adolescent athletes at low (−1 SD) and high (+1 SD) levels of fear of negative evaluation.

The moderation effect is shown in Figure 3. This figure presents the interaction effect of fear of negative evaluation on the relationship between social media exposure and self-compassion. The plotted lines demonstrate that adolescents with high levels of fear of negative evaluation show a steeper negative slope, meaning that their self-compassion decreases more sharply as social media exposure increases. Conversely, those with low fear of negative evaluation display a flatter slope, indicating that their self-compassion is less affected by exposure. This pattern suggests that fear of negative evaluation amplifies vulnerability to social media influences, thereby making adolescents more prone to diminished self-compassion. At the same time, the figure also illustrates the protective side: when fear of negative evaluation is low, social media exposure has a relatively weaker negative impact, showing the conditional nature of the mediated pathway.

## 5 Discussion

The present study examined how social media exposure was associated with body image dissatisfaction among adolescent athletes by testing the mediating role of self-compassion and the moderating role of fear of negative evaluation. The findings provide empirical support for all proposed hypotheses and offer several insights when interpreted in relation to existing literature.

First, consistent with H1, social media exposure was positively associated with body image dissatisfaction among adolescent athletes. This finding aligns with prior research demonstrating that frequent engagement with appearance-focused social media content intensifies upward social comparisons and negative body evaluations among adolescents (Alruwayshid et al., 2021; Jiotsa et al., 2021; Li et al., 2024). Importantly, the result extends this literature by showing that adolescent athletes—despite their functional body orientation and training experience—remain susceptible to appearance-based pressures in digital environments, echoing recent evidence that athletic participation does not fully buffer against media-driven body concerns (Beos et al., 2025; Webb et al., 2024). Within athletic settings, these appearance pressures may become psychologically intensified because adolescent athletes are evaluated not only on physical attractiveness but also on body functionality, discipline, and competitive legitimacy, thereby exposing them to simultaneous online and offline comparison demands. In this regard, the findings suggest that body image dissatisfaction among adolescent athletes may emerge through the combined influence of media-driven appearance ideals and performance-based evaluative environments.

Second, supporting H2, social media exposure was negatively related to self-compassion. This finding is consistent with studies suggesting that repeated exposure to idealized online content erodes self-kindness and mindful self-acceptance by fostering persistent self-evaluation and comparison (Scott et al., 2021; Staśkiewicz-Bartecka et al., 2024). For adolescent athletes, such exposure may be particularly impactful, as evaluative standards related to both performance and appearance are already salient within sporting contexts (Bjornsen and O’Connor, 2025). Rather than functioning merely as passive media consumption, appearance-oriented social media engagement may reinforce continuous self-monitoring and comparison processes that gradually weaken adolescents’ ability to maintain compassionate self-attitudes under conditions of perceived external scrutiny.

Third, in line with H3, self-compassion was negatively associated with body image dissatisfaction. This result corroborates extensive evidence indicating that individuals with higher self-compassion exhibit greater body appreciation and lower vulnerability to dissatisfaction and disordered eating behaviors (Hooper et al., 2024; Linardon et al., 2022). The present finding confirms that these protective effects are also evident among adolescent athletes, a population that has received comparatively less attention in self-compassion research. The finding further highlights that self-compassion may operate as a psychologically protective resource within highly evaluative sports environments by helping adolescents regulate self-critical responses triggered by appearance-related comparison pressures.

Fourth, the mediation analysis supported H4, demonstrating that self-compassion partially mediated the relationship between social media exposure and body image dissatisfaction. This finding is consistent with prior work showing that self-compassion translates external appearance pressures into internal emotional outcomes by shaping how individuals respond to self-critical thoughts triggered by comparison (Adam et al., 2021; Tylka and Huellemann, 2023). By identifying self-compassion as a mediating mechanism in adolescent athletes, the study extends existing research that has primarily examined general or adult samples (Beos et al., 2025). Because both the direct effect of social media exposure on body image dissatisfaction and the indirect effect through self-compassion remained significant and operated in the same direction, the mediation pattern can be interpreted as complementary mediation (Zhao et al., 2010). Accordingly, the findings suggest that the association between social media exposure and body image dissatisfaction may not operate solely through direct comparison processes, but also through the erosion of adolescents’ capacity for adaptive and compassionate self-regulation. Although the observed effects were moderate in magnitude, they remain practically meaningful within adolescent sport psychology contexts because even modest increases in body dissatisfaction may accumulate over time and influence psychological wellbeing, self-esteem, performance confidence, and coping processes among athletes exposed to continuous evaluative pressures.

Finally, the moderation analysis confirmed H5, revealing that fear of negative evaluation strengthened the negative association between social media exposure and self-compassion. Specifically, adolescent athletes with higher levels of fear of negative evaluation experienced a sharper decline in self-compassion as social media exposure increased. This finding aligns with earlier studies identifying fear of negative evaluation as a dispositional vulnerability that heightens sensitivity to social judgment and comparison cues (Gabrys and Wontorczyk, 2023; Weeks et al., 2005). The result further suggests that adolescent athletes who are more sensitive to external judgment may be especially vulnerable to the adverse psychological consequences of appearance-oriented social media exposure, thereby reinforcing the conditional nature of digital comparison processes (Lin et al., 2025). From a broader theoretical perspective, the findings support the notion of “social evaluation sensitivity,” whereby adolescents with heightened concern regarding external judgment may experience digitally mediated appearance comparisons as psychologically amplified forms of social scrutiny (Gabrys and Wontorczyk, 2023; Weeks et al., 2005). This process may be particularly salient among adolescent athletes because online appearance evaluations coexist with offline performance-based evaluations from coaches, peers, parents, and broader social networks. Within the Chinese collectivist context (Lin et al., 2025), where collective reputation, authority expectations, academic achievement, and sports-special recruitment opportunities often shape adolescents’ future-oriented self-perceptions, sensitivity to external evaluation may become even more psychologically consequential. Accordingly, the findings suggest that body image formation among Chinese adolescent athletes may reflect the interaction of vulnerability factors, such as fear of negative evaluation, and protective resources, such as self-compassion, within highly evaluative digital and sociocultural environments.

### 5.1 Theoretical implications

The findings of this study provide several important theoretical implications that extend existing knowledge in meaningful ways. More broadly, the study contributes to a more integrated understanding of how body image dissatisfaction among adolescent athletes may emerge through the interaction of vulnerability and protective psychological processes operating within digitally saturated and highly evaluative environments. Rather than viewing social media exposure as an isolated influence, the present findings suggest that body image dissatisfaction may reflect a contextually embedded process shaped by appearance-based comparison pressures, adolescents’ sensitivity to external evaluation, and their capacity for compassionate self-regulation (Festinger, 1954; Neff, 2003; Gabrys and Wontorczyk, 2023). In this regard, self-compassion appears to function as a protective psychological resource that may reduce the internalization of media-driven appearance ideals, whereas fear of negative evaluation represents a vulnerability factor that may intensify adolescents’ sensitivity to both online and offline evaluative pressures (Linardon et al., 2022; Weeks et al., 2005).

To begin with, the positive association observed between social media exposure and body image dissatisfaction among adolescent athletes reinforces earlier work which has consistently linked social media use to negative body evaluations (Alruwayshid et al., 2021; Jiotsa et al., 2021). Yet, in contrast to prior studies largely conducted in Western or general adolescent samples (e.g., Adam et al., 2021; Izydorczyk et al., 2023), by situating this relationship in the context of Chinese adolescent athletes, the study broadens the scope of Social Comparison Theory (Festinger, 1954), showing that comparison processes are not confined to Western samples or non-athletic populations but are equally influential in athletic subcultures where performance and physique are central. In doing so, the study responds to calls for more culturally and contextually grounded examinations of media effects (Möri et al., 2022; Vandenbosch et al., 2022) and extends the literature by highlighting that athletes, despite their training and functional focus, remain vulnerable to the appearance-based comparisons that social media promotes. Importantly, the findings further suggest that adolescent athletes may experience a distinct form of dual evaluative pressure because they are simultaneously exposed to media-driven appearance ideals and performance-oriented bodily expectations embedded within sports environments (Bjornsen and O’Connor, 2025; Hardie et al., 2022). Such overlapping pressures may intensify adolescents’ tendency to evaluate their bodies not only in terms of attractiveness but also in relation to discipline, competence, and athletic legitimacy.

Equally important are the findings regarding self-compassion, which emerged as a significant mediator of the link between social media exposure and body image dissatisfaction. Prior research has highlighted self-compassion as a protective factor against negative body image outcomes (Castellanos Silva and Steins, 2023), but existing studies have primarily examined this relationship in isolation or as a direct association, rather than as part of an integrated explanatory process. The present study advances Self-Compassion Theory (Neff, 2003) by empirically demonstrating that lower levels of self-compassion explain why heightened social media exposure is associated with dissatisfaction among adolescent athletes. By formally testing self-compassion as a mediating mechanism within a broader process model, this study extends prior work such as Adam et al. (2021), which did not examine how protective psychological resources operate within a conditional pathway. In contrast to earlier studies conducted mainly in general or clinical samples, this work provides evidence from a performance-oriented adolescent group, suggesting that compassionate self-attitudes are equally crucial in sports contexts where external pressures are often amplified. Accordingly, the findings imply that self-compassion may not simply buffer emotional distress after comparison occurs, but may also shape how adolescents cognitively interpret and emotionally regulate appearance-related evaluations within digital environments (Mahon and Hevey, 2023; Tylka and Huellemann, 2023).

The moderating role of fear of negative evaluation offers another theoretical contribution. Earlier studies have recognized fear of negative evaluation as a vulnerability factor in social anxiety and body image concerns (Gabrys and Wontorczyk, 2023; Weeks et al., 2005), but its role in shaping the indirect process through which social media exposure relates to body image dissatisfaction has remained largely unexplored. By demonstrating that adolescents with higher levels of fear of negative evaluation experience a steeper reduction in self-compassion under conditions of elevated social media exposure, the study introduces fear of negative evaluation as a boundary condition within an integrated moderated mediation framework. This finding extends Social Comparison Theory by illustrating that comparison outcomes are not uniform but depend on individual dispositional sensitivities, and it also enriches the self-compassion literature by demonstrating that its protective function is contingent upon evaluative concerns—an interaction not explicitly addressed in earlier models. More specifically, the findings suggest that adolescents with heightened concern regarding external judgment may experience appearance-oriented social media environments as psychologically intensified spaces of social scrutiny, thereby increasing vulnerability to self-critical evaluation processes (Gabrys and Wontorczyk, 2023; Lin et al., 2025).

Taken together, the study presents a comprehensive model that integrates environmental exposure, psychological resources, and individual vulnerabilities to explain body image dissatisfaction in adolescent athletes. Distinct from prior literature, the present research simultaneously accounts for mediation and moderation processes while embedding these mechanisms within a collectivist cultural context. The inclusion of Chinese adolescent athletes broadens the cultural scope of body image research, the moderated mediation design clarifies how and under what conditions social media exposure is associated with dissatisfaction, and the cultural framing highlights how body-related evaluations are intertwined with future-oriented pressures linked to achievement, legitimacy, and long-term prospects. Within the Chinese collectivist context, adolescents’ self-worth may be more strongly connected to external evaluation, parental expectations, coach authority, peer comparison, and collective reputation, all of which may amplify the psychological salience of appearance- and performance-related judgments (Lin et al., 2025). Moreover, because athletic achievement in China is often connected to educational mobility and sports-special recruitment opportunities, body-related evaluations may carry broader symbolic implications associated with discipline, future success, and social recognition. In this way, the study contributes to a more culturally embedded understanding of body image formation among adolescent athletes by demonstrating how digital comparison processes interact with broader sociocultural and achievement-oriented evaluative systems.

### 5.2 Practical implications

The findings of this study also yield meaningful practical implications for educators, coaches, parents, and policymakers concerned with adolescent athletes’ wellbeing. First, the evidence that social media exposure was positively associated with body image dissatisfaction highlights the need for awareness programs that encourage critical engagement with online content. Coaches and teachers can integrate media literacy sessions into training or school curricula, enabling adolescents to recognize unrealistic portrayals of bodies and potentially reduce the psychological impact of harmful upward comparisons. Because adolescent athletes are simultaneously exposed to online appearance ideals and offline performance evaluations, interventions may be particularly important in sports environments where body-related scrutiny is normalized (Bjornsen and O’Connor, 2025; Hardie et al., 2022).

Second, the mediating role of self-compassion suggests that interventions aimed at strengthening compassionate self-attitudes may help buffer the negative psychological experiences associated with appearance-oriented social media engagement. Practical approaches such as mindfulness-based practices, reflective journaling, or guided compassion exercises have been associated with greater emotional resilience and may therefore be adapted into athletic training programs to help adolescents cope with both performance pressures and media-related evaluative concerns (Mahon and Hevey, 2023; Tylka and Huellemann, 2023). In highly evaluative athletic contexts, fostering self-compassion may help adolescents interpret body-related criticism in less self-destructive ways and reduce excessive self-monitoring tendencies associated with social comparison processes.

The moderating effect of fear of negative evaluation further suggests that prevention and intervention strategies should be tailored to adolescents who are especially sensitive to peer and authority-based judgment. Screening tools may be used by school counselors or sports psychologists to identify athletes with elevated fear of negative evaluation, allowing for more targeted psychological support. Group discussions and workshops that normalize imperfection and reduce evaluative anxieties may also help mitigate vulnerability among adolescents exposed to appearance-focused digital environments. Within the Chinese collectivist context, where parental expectations, coach authority, collective reputation, and future educational opportunities may strongly shape adolescents’ self-worth, reducing evaluative pressure within sports settings may be especially important for protecting psychological wellbeing (Lin et al., 2025).

Importantly, these practical applications extend beyond individual coping strategies to institutional responsibilities. Sports organizations and schools can foster more supportive environments where appearance-based criticism is minimized and greater emphasis is placed on health, resilience, skill development, and overall wellbeing rather than idealized appearance standards alone. By aligning the findings with actionable strategies, the study underscores the importance of comprehensive support systems that may help safeguard adolescent athletes from the psychological risks associated with appearance-oriented social media engagement.

### 5.3 Limitations

This study is not without limitations, which should be acknowledged when interpreting the findings. First, the use of a cross-sectional design restricts the ability to draw causal inferences, as relationships among variables were observed at a single point in time. Future research employing longitudinal or experimental designs would help establish temporal order and causality.

Second, reliance on self-reported measures may have introduced social desirability bias, particularly among athletes who might underreport dissatisfaction or evaluative fears. Although procedural and statistical remedies were applied to reduce common method bias, such bias cannot be entirely ruled out when data are collected from a single source. In addition, Harman’s single-factor test, while widely used, has recognized methodological limitations when employed as a standalone assessment of common method variance. Future studies may therefore benefit from incorporating more advanced approaches, such as marker-variable or latent-method-factor techniques, to further strengthen methodological rigor.

Third, the sample was limited to adolescent athletes in China, which, while offering cultural and contextual insight, may restrict the generalizability of findings to other populations or cultural contexts where media use patterns, athletic structures, and body ideals differ. Moreover, although the study employed a multistage sampling strategy across multiple provinces, the participating institutions were selected partly based on accessibility and the availability of organized sports programs. Accordingly, the findings may not fully represent adolescent athletes from regions or institutional settings with different athletic structures, sociocultural conditions, or levels of sports participation.

In addition, although the study conceptualized social media exposure primarily as engagement intensity within appearance-oriented digital environments, one measurement item also reflected comparison-oriented engagement, which may indicate partial conceptual overlap between exposure and social comparison processes. Future research may therefore benefit from distinguishing more explicitly between passive exposure, active comparison behavior, and appearance-focused cognitive engagement within social media environments.

Finally, the study did not account for potential moderating influences such as gender differences, type of sport, or socioeconomic background, which may further nuance the observed relationships. Addressing these limitations in future research would enhance the robustness and broader applicability of the findings.

## 6 Conclusion

In conclusion, this study provides empirical evidence that social media exposure was positively associated with body image dissatisfaction among adolescent athletes, both directly and indirectly through reduced self-compassion, while fear of negative evaluation strengthened the negative association between social media exposure and self-compassion. Grounded in Social Comparison Theory and Self-Compassion Theory, the findings highlight the dual role of protective and vulnerability factors in shaping adolescents’ responses to media-driven appearance comparisons. By focusing on Chinese adolescent athletes, the study extends existing literature to a performance-oriented and culturally distinct population, thereby broadening theoretical and practical understanding of body image formation within highly evaluative digital and athletic environments.

More specifically, the findings suggest that body image dissatisfaction among adolescent athletes may emerge through the interaction of digitally mediated comparison pressures, heightened sensitivity to external evaluation, and adolescents’ capacity for compassionate self-regulation. Within the Chinese collectivist context, these processes may become particularly salient because body-related evaluations are often intertwined with broader expectations concerning achievement, discipline, collective reputation, and future opportunity structures. Overall, the findings underscore the importance of fostering self-compassion and reducing excessive evaluative pressure within adolescent sports environments while offering valuable directions for future research and intervention development aimed at supporting adolescent athletes’ psychological wellbeing.

## Data Availability

The original contributions presented in the study are included in the article/Supplementary material, further inquiries can be directed to the corresponding author.
