# Supplementary material for: Investigating the relationship between social media exposure, body image dissatisfaction, and self-compassion in adolescent athlete
Source: Front Psychol. 2026 Jun 5;17:1819872. doi: 10.3389/fpsyg.2026.1819872 (PMC13278878; doi:10.3389/fpsyg.2026.1819872)
Supplement: Supplementary file 1 [file Table_1.docx]

### ****Appendix 1.** Demographic Characteristics of the Sample (N = 449)**

| **Variable** | **Category** | **n** | **%** |
| --- | --- | --- | --- |
| **Gender** | Male | 245 | 54.6 |
|  | Female | 204 | 45.4 |
| **Age (years)** | 13–15 | 171 | 38.1 |
|  | 16–18 | 278 | 61.9 |
| **School Level** | Middle school | 190 | 42.3 |
|  | High school | 259 | 57.7 |
| **Sport Type** | Aesthetic sports (e.g., gymnastics, dance) | 131 | 29.2 |
|  | Non-aesthetic sports (e.g., basketball, athletics, badminton) | 318 | 70.8 |
| **Training Experience** | < 2 years | 101 | 22.5 |
|  | 2–4 years | 184 | 41.0 |
|  | > 4 years | 164 | 36.5 |
| **Weekly Training Hours** | < 5 hours | 85 | 18.9 |
|  | 5–9 hours | 212 | 47.2 |
|  | ≥ 10 hours | 152 | 33.9 |
| **Daily Social Media Use** | < 1 hour | 69 | 15.4 |
|  | 1–3 hours | 220 | 49.0 |
|  | > 3 hours | 160 | 35.6 |
| **Region** | Eastern China | 199 | 44.3 |
|  | Central China | 122 | 27.2 |
|  | Western China | 128 | 28.5 |

## ****Appendix 2.** Measurement Items**

### ****Social Media Exposure****

1. I often compare myself with others on social media.
2. I spend a lot of time browsing content on social media platforms.
3. I frequently check social media updates during the day.
4. I am regularly exposed to posts or images shared by others on social media.
5. I use social media platforms as part of my daily routine.
6. I pay close attention to posts shared by peers on social media.
7. I feel highly engaged when using social networking sites.

### ****Self-Compassion****

1. I try to be understanding and patient toward aspects of my personality I do not like.
2. When I fail at something important to me, I try to keep things in perspective.
3. I am kind to myself when I experience difficulties.
4. I try to see my struggles as part of being human.
5. I give myself the care and tenderness I need during tough times.
6. I try not to be overly critical of myself when things go wrong.
7. I approach my personal shortcomings with balance and acceptance.
8. I remind myself that everyone makes mistakes sometimes.

### ****Body Image Dissatisfaction****

1. I often feel unhappy with the way my body looks.
2. I am dissatisfied with my body shape.
3. I feel uncomfortable with my physical appearance.
4. I wish my body looked different from how it does now.
5. I feel negative about my appearance when I see myself in the mirror.
6. I am unhappy with specific parts of my body.
7. I feel dissatisfied with how my body appears to others.
8. I feel that my appearance does not meet my expectations.
9. I am often critical of my body image.

### ****Fear of Negative Evaluation****

1. I worry about what other people think of me even when I know it doesn’t matter.
2. I am afraid that others will find fault with me.
3. I worry about being judged negatively by others.
4. I am concerned about making a bad impression on people.
5. I feel anxious when I think others are evaluating me.

### Appendix 3. Confirmatory Factor Analysis: Model Fit Indices

| **Fit Index** | **Recommended Cutoff** | **CFA Model Value** | **Interpretation** |
| --- | --- | --- | --- |
| χ² (Chi-square) | — | 742.36 | — |
| df | — | 341 | — |
| χ² / df | < 3.00 | 2.18 | Good fit |
| CFI (Comparative Fit Index) | ≥ .90 (>.95 ideal) | .942 | Good fit |
| TLI (Tucker–Lewis Index) | ≥ .90 | .931 | Good fit |
| RMSEA (Root Mean Square Error of Approximation) | ≤ .08 (≤ .06 ideal) | .057 | Acceptable fit |
| SRMR (Standardized Root Mean Square Residual) | ≤ .08 | .046 | Good fit |

**Appendix 4.** Preliminary demographic comparisons across main study variables

| **Demographic Variable** | **Statistical Test** | **Main Constructs Examined** | **Significant Difference** | **Interpretation** |
| --- | --- | --- | --- | --- |
| Gender | Independent-samples t-test | BID, SC, SME, FNE | No consistent significance | No substantive influence on structural paths |
| School Level | One-way ANOVA | BID, SC, SME, FNE | Marginal differences observed | Did not alter hypothesized relationships |
| Sport Type | Independent-samples t-test | BID, SC, SME, FNE | Minor differences observed | Effects not substantively meaningful |
| Training Experience | Correlation analysis | BID, SC, SME, FNE | Weak correlations | No meaningful confounding effect |
| Weekly Training Hours | Correlation analysis | BID, SC, SME, FNE | Weak correlations | No meaningful confounding effect |
| **Note:** BID = Body Image Dissatisfaction; SC = Self-Compassion; SME = Social Media Exposure; FNE = Fear of Negative Evaluation. | | | | |
